# The prevalence of cardiac complications and their impact on outcomes in patients with non-traumatic subarachnoid hemorrhage

**DOI:** 10.1038/s41598-022-24675-8

**Published:** 2022-11-22

**Authors:** Maarit Lång, Stephan M. Jakob, Riikka Takala, Magnus N. Lyngbakken, Anu Turpeinen, Torbjørn Omland, Tobias M. Merz, Jan Wiegand, Juha Grönlund, Melissa Rahi, Mika Valtonen, Timo Koivisto, Helge Røsjø, Stepani Bendel

**Affiliations:** 1 Department of Intensive Care Medicine, Kuopio University Hospital, University of Eastern Finland, PO BOX 100, 70029 KYS Kuopio, Finland; 2 Department of Intensive Care Medicine, University Hospital of Bern, University of Bern, Bern, Switzerland; 3 Perioperative Services, Intensive Care Medicine and Pain Management, Turku University Hospital, Anaesthesiology, Intensive Care, Emergency Care and Pain Medicine, University of Turku, Turku, Finland; 4 Division of Medicine, Department of Cardiology, Akershus University Hospital, Lørenskog, Institute of Clinical Medicine, University of Oslo, Oslo, Norway; 5 Department of Cardiology, Kuopio University Hospital, University of Eastern Finad, Kuopio, Finland; 6 Neurocenter, Department of Neurosurgery, Turku University Hospital, University of Turku, Turku, Finland; 7 Department of Neurosurgery, Kuopio University Hospital, University of Eastern Finland, Kuopio, Finland; 8 Division for Research and Innovation, Akershus University Hospital, Lørenskog, Institute of Clinical Medicine, University of Oslo, Oslo, Norway; 9 Present Address: Cardiothoracic and Vascular Intensive Care Unit, Auckland City Hospital, Auckland, New Zealand; 10 Present Address: Intensive Care Unit, Lindenhofspital, Bern, Switzerland

**Keywords:** Cardiology, Medical research, Neurology, Risk factors, Signs and symptoms

## Abstract

Subarachnoid hemorrhage (SAH) is a serious condition, and a myocardial injury or dysfunction could contribute to the outcome. We assessed the prevalence and prognostic impact of cardiac involvement in a cohort with SAH. This is a prospective observational multicenter study. We included 192 patients treated for non-traumatic subarachnoid hemorrhage. We performed ECG recordings, echocardiographic examinations, and blood sampling within 24 h of admission and on days 3 and 7 and at 90 days. The primary endpoint was the evidence of cardiac involvement at 90 days, and the secondary endpoint was to examine the prevalence of a myocardial injury or dysfunction. The median age was 54.5 (interquartile range [IQR] 48.0–64.0) years, 44.3% were male and the median World Federation of Neurological Surgeons (WFNS) score was 2 (IQR 1–4). At day 90, 22/125 patients (17.6%) had left ventricular ejection fractions ≤ 50%, and 2/121 patients (1.7%) had evidence of a diastolic dysfunction as defined by mitral peak E-wave velocity by peak eʹ velocity (E/eʹ) > 14. There was no prognostic impact from echocardiographic evidence of cardiac complications on neurological outcomes. The overall prevalence of cardiac dysfunction was modest. We found no demographic or SAH-related factors associated with 90 days cardiac dysfunction.

## Introduction

A subarachnoid hemorrhage (SAH) is a serious condition with high mortality and morbidity. Patients with SAH may develop several complications, including acute myocardial injury and cardiac dysfunction^1^. Accordingly, there is a need for updated evidence and information on the prevalence of cardiac injury, myocardial dysfunction, and cardiac arrhythmias in a contemporary cohort of SAH patients. More information is also needed regarding the relationship between cardiac involvement and clinical outcomes in SAH patients.

A left ventricle (LV) dysfunction occurs most often in SAH patients with elevated cardiac enzymes and B-type natriuretic peptides (BNP)^2^, electrocardiogram changes (ECG) and severe grades of SAH^3^. The triad of elevated cardiac biomarkers, ventricular arrhythmias^4^ and eventually overt cardiac dysfunction^5–7^ has been observed for decades in SAH patients^6^, but whether they represent risks in addition to established risk models is currently not known. Moreover, to detect cardiac involvement in patients with SAH, there is a need to integrate information from ECG, cardiac biomarkers, and echocardiography^4^. Accordingly, in this multicenter epidemiological study, we aimed to provide updated information related to evidence of cardiac involvement in non-traumatic SAH patients and to examine the prognostic impact of myocardial injury or dysfunction added to established risk scoring systems.

The study is registered in Clinical Trials (NCT01670838) 22/08/2012.

## Methods

### Participants

We included 197 consecutive patients treated in Kuopio University Hospital, Finland, Turku University Hospital, Finland, and Bern University Hospital, Switzerland, from March 2014 to February 2016. Five patients were excluded due to the missing World Federation of Neurological Surgeons (WFNS) score. The inclusion criteria were patients with acute non-traumatic SAH, age ≥ 18 years and written consent. The exclusion criteria were anticipated brain death < 24 h or an otherwise moribund patient (expected to die < 24 h or treated only as a donor candidate). Screening log is presented below.
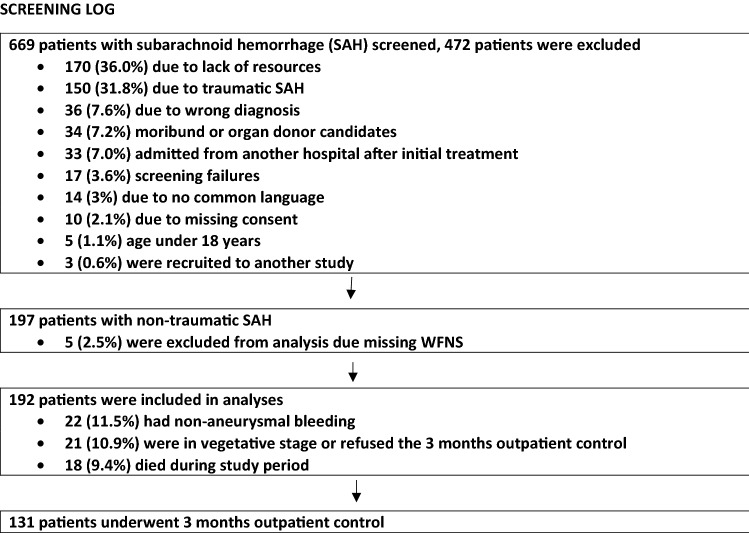


Written informed consent was requested from the patients by the intensive care unit (ICU) study personnel. If the patient was not capable of acting, consent was requested from next-of-kin or the patient’s legal representative. This manuscript reports results that were acquired according to the Strengthening the Reporting of Observational Studies in Epidemiology (STROBE) guidelines. Five patients with missing data for World Federation of Neurological Surgeons (WFNS) scores were excluded, leaving 192 patients available for analyses.

All measurements were made during ICU and hospital stays, and the 90-day measurements were performed at the outpatient visit. Systolic cardiac dysfunction was defined as a left ventricular ejection fraction (LVEF) ≤ 50%, and diastolic dysfunction was defined as a ratio of early mitral inflow velocity, and mitral annular early diastolic velocity (E/eʹ) > 14 by echocardiography. The severity of SAH was classified using the WFNS score as follows: grade I Glasgow coma scale (GCS) 15, no motor deficit, grade II GCS 13–14, no motor deficit, grade III GCS 13–14 and motor deficit, grade IV GCS 7–12 and grade V GCS 3–6. The Hunt and Hess clinical grading system was also used to classify the severity of SAH, and we used the Fisher scale to grade the computer tomography appearance of bleeding^8^ (Table 1).

**Table 1:** The severity of bleeding.

**Hunt and Hess scale**	
Grade I	Mild headache and nuchal stiffness, no neurological deficit
Grade II	Moderate headache and nuchal stiffness, no neurologic deficit except possibly a cranial nerve palsy
Grade III	Somnolence, possibly mild focal neurologic deficit
Grade IV	Stupor, hemiparesis (moderate to severe)
Grade V	Coma
**Fisher scale**	
Grade 0	No scan available
Grade 1	No blood detected
Grade 2	Diffuse deposition or thin layer. All vertical layers of blood < 1 mm thick
Garde 3	Localized clots and/or vertical layers of blood > 1 mm thick
Grade 4	Intraventricular or intra parenchymal blood present

The intensive care treatment protocol of the SAH patients is presented in Table 2.

**Table 2:** The intensive care treatment protocol.

Positioning	Head up tilt 30°, No rotation, flexion, or extension of head
Systolic blood pressure	< 140–160 mmHg before clipping/coiling of aneurysm Normotension after aneurysm treatment or hypertension on clinical basis
Intracranial pressure	< 15–20 mmHg
Cerebral perfusion pressure	> 60 mmHg Volume to CVP 2–8 mmHg / normovolemia Norepinephrine-infusion Lowering the dose of intravenous nimodipine
Sedation on clinical basis	Propofol-infusion: Aim at RASS scale 0–5 depending on ICP Bolus when needed (nursing, suction etc.) Opioid-boluses: Muscle relaxation if needed
Ventilator treatment	PaCO2 30.0–33.8 mmHg, PaO2 > 97.5 mmHg, SpO2 > 95%
Temperature	< 37.5 °C
Hb, throm, INR/TT%	> 10.0 g/dL, > 100 *10^3^/mm^3^, < 1.5 or > 60%
Electrolytes	S-Na > 140 mEq/L, S-Mg at normal range
Infections	Treated on clinical basis
Nutrition	Routine protocol with early enteral feeding
Thrombosis prophylaxis	Enoxaparin or heparin if no risk of rebleeding Intermittent pneumatic compression stockings and or antiembolia stockings if enoxaparin cannot be used
Cerebral vasospasm and symptoms of delayed cerebral ischemia	Blood pressure and perfusion pressure management was tailored individually Angioplasty or intra-arterial spasmolytics

*°C* degrees Celsius, *CVP* central venous pressure, *Hb* hemoglobin, *ICP* intracranial pressure, *INR* international standardized ratio, *mmHg* millimeters of mercury, *PaCO2* partial pressure of carbon dioxide, *PaO2* partial pressure of oxygen pressure, *RASS* Richmond agitation sedation scale, *SpO2%* peripheral capillary oxygen saturation percent, *Throm* thrombocytes, *TT%* thromboplastin time.

### Data collection

Patient demographics were collected prospectively from electronic patient data management systems, including the admission WFNS score^9^. Routine laboratory markers were collected daily at 8 a.m. from admission to day 7 and at 90 days at the outpatient clinic. We collected the following routine laboratory markers: blood gases, blood hemoglobin, thrombocytes, leukocytes, international normalized ratio (INR), bilirubin, creatinine, C-reactive protein (CRP), creatinine kinase (CK), creatinine kinase myocardial band (CK-MB), cardiac troponin T (cTnT), N‐terminal pro‐B‐type natriuretic peptide (NT-proBNP), sodium, potassium, and magnesium. All routine laboratory samples were analyzed by accredited laboratories at the study hospitals. Both cTnT and NT-proBNP were measured by the electrochemiluminescence immunoassay (ECLIA) assays (Roche Diagnostics GmbH, Mannheim, Germany). We calculated the estimated glomerular filtration (eGFR) using the CKD-EPI formula^10^.

We also measured plasma norepinephrine and epinephrine concentrations during the first 24 h after admission (day 1) and later at days 3 and 7 and after 3 months. For these measurements, plasma samples were collected in 10 ml plastic tubes in ice containing EGTA (Ethylene glycolbis (2-aminoethylether)-N, N, Nʹ, Nʹ-tetra acetic acid) and reduced glutathione as a preservative. The samples were centrifuged immediately, and the plasma was stored frozen at − 70 °C until analyzed. For the chromatographic analysis of catecholamines, a Chromsystems reagent kit (Chromsystems Instruments and Chemicals GmbH, Munich, Germany) was used. Body mass index (BMI) was calculated by weight (Kg)/ [height (m)] ^2^.

Transthoracic echocardiography (TTE) was performed during the first 24 h after admission (day 1), at days 3 and 7 and at 3 months outpatient clinic visit. A limited number of experienced cardiologists or intensivists performed cardiac ultrasound examinations according to the specific study protocol. LV end-diastolic diameter (LVEDD), end-systolic diameter (LVESD), interventricular wall thickness (IVS), posterior wall thickness (PW), aortic root diameter (Ao) and left atrium diameter (LA) were recorded from the parasternal long-axis view. LVEF was measured using the parasternal M-mode view and apical 4-chamber projection and was calculated using the Simpson method. We assessed motion abnormalities in the anterior, lateral, inferior and septal walls using the long-axis parasternal view, and the findings were reported as normal wall motion/hypokinesia/akinesia/dyskinesia. LV diastolic function was assessed based on the mitral inflow pattern, E-wave and A-wave velocities, E/A ratio and deceleration time (DT). Diastolic tissue motion velocity in the lateral mitral annulus (lat eʹ) and septal annulus (sept eʹ) was recorded using pulsed wave tissue Doppler and was averaged (eʹ). Diastolic function and left ventricular filling pressure were assessed by calculating the E/eʹ ratio. Right ventricular function was assessed by a tricuspid annulus plane systolic excursion (TAPSE) and by measuring systolic tissue velocity in the tricuspid annulus (tricuspid Sʹ). The right ventricular end-diastolic diameter was measured from the apical 4-chamber view. Pulmonary artery pressure was estimated based on tricuspid regurgitation. Other significant abnormalities of the heart (e.g., valves, pericardial effusion, atrial septal defect, intracardial thrombosis) were also recorded.

We performed a 24-h Holter®-monitoring at day 1 and at day 7, concurrent with the cardiac echocardiography examination, and we performed Holter-monitoring at the 3-month outpatient visit. The Holter-registrations were performed using a Medilog AR4-recorder. Data were automatically analyzed by a software engine (Darwin, ScanMed AS) with manual corrections for artefacts. The mean heart rate and any arrhythmias were registered as well as measures of heart rate variability (standard deviation of RR-interval (SDNN), power in the high-frequency spectrum, power in the low-frequency spectrum and their ratios.

### Clinical outcomes

All patients were scheduled for a routine 90-day neurosurgical follow-up, and the neurologic outcome was assessed using the Modified Rankin Scale (mRS)^11^ or the Glasgow Outcome Scale (GOSE)^11^. We did not have three-month follow-ups for patients with non-aneurysmal bleeding (n = 22) or patients with no need for clinical control due to a vegetative state. For the prognostic analyses, we dichotomized the outcome as a good clinical outcome, which we defined as mRS 0–2 or GOSE 6–8, or a poor clinical outcome for patients that died or were dependent on help after SAH (mRS > 2 or GOSE < 6). The definitions of cardiac complications during admission and follow-up are detailed in Table 3.

**Table 3:** Definitions of cardiac and other complications.

Elevated cTnT	≥ 14 ng/L
Elevated NT-proBNP	> 450 ng/L (< 50 years)
	> 900 ng/L (50–75 years)
	> 1800 ng/L (> 75 years)
Rhythm disturbances on ECG	Atrial fibrillation, premature ventricular contractions, supraventricular extrasystole, AV-block
Ischemia on ECG	ST elevation, ST depression. T inversion
QTc prolongation	> 440 ms

*AV* atrioventricular, *cTnT* cardiac troponin T, *NT-proBNP* N‐terminal pro‐B‐type natriuretic peptide, *QTc* corrected QT interval.

### Statistical analysis

This was a prospective observational study investigating the incidence of cardiac involvement with the aim to document possible predisposing factors during ICU stays for cardiac dysfunction at 90 days in patients with acute non-traumatic SAH. Power calculations before study commencement demonstrated that a sample size of 200 patients would be sufficient to detect a weak correlation (r = 0.20) with alpha 0.05 and power 80%, and we based patient inclusion on this calculation. This sample size would enable group comparisons with an adequate power and with this cohort size also the regression analysis could be performed reliably. Sample size calculations were executed by R statistical software with library ‘pwr’. Categorical data are presented as absolute numbers (proportions) and continuous data as the median (interquartile range [IQR]). For categorical variables, the two-sided χ^2^ test or Fischer’s exact test were used. Continuous data were compared with the Mann–Whitney U-test or the Kruskal–Wallis test of variance.

First, we aimed to assess the prevalence and predictive factors of cardiac complications in patients with non-traumatic SAH. As a secondary outcome measure, we assessed mortality and morbidity caused by cardiac complications. We assessed clinical variables associated with outcomes, and variables with a p-value < 0.10 were included in a multivariable logistical regression model. To determine the association between cardiac involvement and neurological outcomes, we further established a prognostic model with patients stratified into categories based on WFNS grading scores and concentrations of cTnT or NT-proBNP (category 1: WFNS < 3, cTnT < 8 ng/L/NT-proBNP < 380 ng/L [cohort medians], category 2: WFNS < 3, cTnT ≥ 8 ng/L/NT-proBNP ≥ 380 ng/L, category 3: WFNS ≥ 3, cTnT < 8 ng/L/NT-proBNP < 380 ng/L, category 4: WFNS ≥ 3, cTnT ≥ 8 ng/L/NT-proBNP ≥ 380 ng/L). The prognostic models were adjusted for age and sex as well as for a priori selected variables associated with cardiovascular prognosis (systolic blood pressure, BMI, coronary artery disease, diabetes mellitus, current smoking, eGFR and concentrations of norepinephrine). Participants with missing covariate data were excluded from the multivariable regression analyses. The incremental prognostic value of cTnT and NT-proBNP to the WFNS grading score was assessed using C statistics derived from logistic regression models as well as the continuous net reclassification index (cNRI) and integrated discrimination improvement (IDI).

P-values ≤ 0.05 were set to indicate statistically significant results. We used SPSS Statistics for Windows (version 22, IBM Corp, Armonk, NY, USA) and STATA 16.1 (StataCorp LP, College Station, TX) for the statistical analyses.

### Ethical approval

The ethics committees of Northern Savo, Finland (record no 78/2011), Hospital District of Southwest Finland, Turku (T4/2014) and Inselspital Bern, Switzerland (record no 239/12), approved the study. Informed consent was obtained. The study has been performed in accordance with the ethical standards of the 1964 Declaration of Helsinki and its later amendment and is registered in clinical trials 22/08/2012, NCT01670838.

## Results

### Baseline characteristics

The baseline characteristics of the patients according to the WFNS grading scores are presented in Supplement Table S1.

The median age was 54.5 (48.0–64.0) years, 44.3% % were male and the median WFNS was 2 (1–4). In general, the prevalence of premorbid conditions was low. Concentrations of cTnT and norepinephrine as well as QTc increased in parallel with the WFNS score.

### Cardiac complications at admission and at the 90-day follow-up

The details regarding cardiac involvement and other complications during admission and after discharge are outlined in Table 4.

**Table 4:** Cardiac and other complications during admission and after discharge.

	Time point							
	Day 1		Day 3		Day 7		Day 90	
**Cardiac biomarkers**								
Elevated cTnT, n (%)	n = 170	50 (29.4%)	n = 174	51 (29.3%)	n = 133	34 (25.6%)	n = 130	10 (7.7%)***
Elevated NT-proBNP, n (%)	n = 168	43 (25.6%)	n = 174	46 (26.4%)	n = 133	21 (15.8%)*	n = 129	4 (3.1%)***
**ECG**								
Any rhythm disturbance, n (%)	n = 192	27 (14.1%)	n = 192	21 (10.9%)	n = 192	14 (7.3%)*	n = 131	13 (9.9%)
Signs of ischemia, n (%)	n = 192	16 (8.3%)	n = 192	19 (9.9%)	n = 192	16 (8.3%)	n = 130	7 (5.4%)
Any rhythm disturbance or ischemia, n (%)	n = 192	40 (20.8%)	n = 192	36 (18.8%)	n = 192	26 (13.5%)	n = 131	19 (14.5%)
QTc > 440 ms, n (%)	n = 157	88 (56.1%)	n = 177	62 (35.0%)***	n = 134	40 (29.9%)***	n = 130	43 (33.1%)***
First degree AV block, n (%)	n = 185	32 (17.3%)	n = 123	25 (20.3%)	NA		NA	
**Echocardiography**								
LVEF ≤ 50%, n (%)	n = 171	12 (7.0%)	n = 162	17 (0.5%)	n = 145	9 (6.2%)	n = 125	22 (17.6%)**
E/e' > 12, n (%)	n = 178	7 (3.9%)	n = 178	16 (9.0%)	n = 144	5 (3.5%)	n = 121	2 (1.7%)
TAPSE < 15 mm, n (%)	n = 182	3 (1.6%)	n = 179	3 (1.7%)	n = 148	0 (0%)	n = 131	2 (1.5%)
Regional wall motion disturbance, n (%)	n = 192	8 (4.2%)	n = 182	7 (3.6%)	n = 192	4 (2.1%)	n = 131	0 (0%)**
**Radiography**								
Suspected pneumonia, n (%)	n = 80	8 (10.0%)	n = 55	14 (25.5%)*	n = 35	5 (14.3%)	NA	
Congestion, n (%)	n = 80	1 (1.3%)	n = 55	4 (7.3%)	n = 35	0 (0%)	NA	

*AV* atrioventricular, *cTnT* cardiac troponin T, *ECG* electrocardiogram, *LVEF* left ventricular ejection fraction, *NT-proBNP* N‐terminal pro‐B‐type natriuretic peptide, *QTc* corrected QT interval, *TAPSE* tricuspid annulus plane systolic excursion.

p compared to day 1: * < 0.05, ** < 0.01, *** < 0.001.

At day 90, 22/125 patients (17.6%) had LVEF ≤ 50%, and 2/121 patients (1.7%) had E/eʹ > 14. None of the patient population or SAH related investigated factors was predictive of cardiac dysfunction at day 90 (Supplement Table S2). The proportion of patients with elevated cTnT was significantly lower at day 90 (7.7%) compared to day 1 (29.4%; p < 0.001). The proportion of patients with elevated NT-proBNP was significantly lower at day 7 (15.8%) and day 90 (3.1%) compared to day 1 (25.6%; p < 0.001).

The most common ECG finding was QTc prolongation, with an incidence of 56.1% at day 1, 35.0% at day 3, 29.9% at day 7 and 33.1% at day 90 (all p < 0.001 compared to day 1). At day 90, there was a higher proportion of patients with reduced LVEF (< 50%) and a lower proportion of patients with regional wall motion abnormalities compared to the baseline.

Concentrations of cTnT at day 1 according to different categories of clinical status, severity of bleeding and categories of cardiopulmonary events and outcome measures are presented in Table 5.

**Table 5:** Cardiac troponin T concentrations at day 1 according to clinical and cardiac status, and neurological outcome at day 90.

	Cardiac troponin T (ng/L)	p
**Clinical status and severity of bleeding on arrival, n = 192**		
**GCS at arrival**		
9–15	7.0 (5.0–12.0)	< 0.001
3–8	54.0 (12.75–261.00)	
**Worst CGS < 24 h**		
9–15	6.0 (5.0–11.0)	< 0.001
3–8	22.00 (9.0–110.0)	
**World federation of neurological surgeons**		
1–2 (no neurological deficits)	6.0 (5.0–11.5)	< 0.01
3–5	19.0 (8.0–101.0)	
**Hunt and Hess**		
1–2 (headache)	6.0 (5.0–10.0)	< 0.01
3 (drowsy)	9.5 (5.0–24.5)	
4–5 (stupor/coma)	19.0 (8.0–101.0)	
**Fisher**		
1–2 (no blood)	5.0 (5.0–8.0)	0.16
3 (clots)	10.0 (5.0–28.0)	
4–5 (diffuse ICH/IVH)	10.0 (5.25–21.25)	
**Cardiac status at day 1, n = 192**		
Normal NT-proBNP	7.0 (5.0–12.50)	< 0.001
Elevated NT-proBNP	19 .0 (7.0–151.0))	
No rhythm disturbances	7.0 (5.0–19.0)	0.44
Rhythm disturbances	6.0 (5–10.0)	
No ischemia	7.0 (5.0–15.0)	0.26
Ischemia	10.0 (5.0–151.0)	
Normal QTc	6.0 (5.0–10.0)	< 0.01
QTc prolongation	10.0 (6.0–26.0)	
LVEF > 50%	7.0 (2.5–13.75)	0.13
LVEF ≤ 50%	13.0 (5.0–42.0)	
No regional wall motion disturbance	7.0 (5.0–15.0)	< 0.001
Regional wall motion disturbance	472.0 (211.0–1515.25)	
**Neurological outcome at Day 90, n = 131**		
Independent	6.0 (5.0–11.75)	< 0.001
Dependent/dead	13.5 (7.0–45.75)	

Mann–Whitney U or Kruskal-Wallis-test.

*cTnT* cardiac troponin T, *GCS* Glasgow coma scale, *LVEF* left ventricular ejection fraction, *NT-proBNP* N‐terminal pro‐B‐type natriuretic peptide, *QTc* corrected QT interval.

Patients with a worse clinical status and more severe bleeding exhibited higher concentrations of cTnT, which was also the case for patients with worse neurological outcomes at the three-month follow-up. The corresponding analyses for NT-proBNP and endogenous catecholamines are presented in Tables 6 and 7.

**Table 6:** NT-proBNP concentrations at day 1 according to clinical and cardiac status, and neurological outcome at day 90.

	NT-proBNP (ng/L)	p
**Clinical status and severity of bleeding, n = 192**		
**GCS at arrival**		
9–15	356.0 (196.0–727.0)	< 0.001
3–8	1058.0 (349.0–1586.5)	
**Worst CGS < 24 h**		
9–15	346.5 (166.3–650.5)	< 0.001
3–8	900.0 (313.0–1366.0)	
**World federation of neurological surgeons**		
1–2 (no neurological deficits)	352.5 (203.8–651.5)	0.02
3–5	4584.0 (237.0–1294.8)	
**Hunt and Hess**		
1–2 (headache)	401.0 (203.0–652.0)	0.16
3 (drowsy)	320.0 (153.5–826.3)	
4–5 (stupor/coma)	766.0 (240.0–1348.0)	
**Fisher**		
1–2 (no blood)	340.0 (195.0–542.0)	0.11
3 (clots)	357.5 (185.3–1059.3)	
4–5 (diffuse ICH/IVH)	565.0 (240.0–910.0)	
**Cardiac status, n = 192**		
Normal cTnT	279.0 (156.0–538.0)	< 0.001
Elevated cTnT	1058.0 (596.0–1721.5)	
No rhythm disturbances	346.5 (164.0–758.8)	0.50
Rhythm disturbances	456.0 (231.5–804.5)	
No ischemia	357.0 (189.5–742.5)	0.28
Ischemia	1348.0 (227.0–2160.0)	
Normal QTc	306.0 (141.0–581.0)	< 0.01
QTc prolongation	548.0 (239.8–959.3)	
LVEF > 50%	357.0 (197.8–734.5)	0.88
LVEF ≤ 50%	435.0 (156.0–925.0)	
No regional wall motion disturbance	340.0 (197.8–764.3)	< 0.01
Regional wall motion disturbance	1926.0 (580.3–8330.8)	
**Neurological outcome, n = 131**		
Independent	356.0 (173.0–643.0)	0.12
Dependent/dead	400.0 (225.8–1150.8)	

*cTnT* cardiac troponin T, *GCS* Glasgow coma scale, *LVEF* left ventricular ejection fraction, *NT-proBNP* N‐terminal pro‐B‐type natriuretic peptide, *QTc* corrected QT interval.

**Table 7:** Endogenous catecholamine concentrations at day 1 according to clinical and cardiac status, and neurological outcome at day 90.

	Epinephrine (nmol/L)	p	Norepinephrine (nmol/L)	p
**Clinical status and severity of bleeding, n = 192**				
**GCS at arrival**				
9–15	0.51 (0.28–0.74)	0.46	2.7 (1.9–3.7)	0.30
3–8	0.43 (0.24–0.66)		2.9 (2.0–3.9)	
**Worst CGS < 24 h**				
9–15	0.49 (0.28–0.74)	0.95	2.6 (1.9–3.7)	0.29
3–8	0.44 (0.28–0.70)		2.9 (2.0–3.9)	
**World federation of neurological surgeons**				
1–2 (no neurological deficits)	0.53 (0.32–0.75)	0.26	2.6 (1.8–3.7)	0.037
3–5	0.42 (0.24–0.70)		3.1 (2.2–4.0)	
**Hunt and Hess**				
1–2 (headache)	0.54 (0.32–0.76)	0.57	2.6 (1.7–3.7)	0.039
3 (drowsy)	0.56 (0.35–0.73)		3.2 (2.7–5.0)	
4–5 (stupor/coma)	0.41 (0.24–0.66)		3.2 (2.5–3.9)	
**Fisher**				
1–2 (no blood)	0.55 (0.35–0.78)	0.029	2.5 (1.6–3.1)	0.10
3 (clots)	0.57 (0.34–0.80)		2.6 (1.7–3.7)	
4–5 (diffuse ICH/IVH)	0.41 (0.22–0.65)		2.9 (2.0–4.0)	
**Cardiac status, n = 192**				
Normal cTnT	0.51 (0.32–0.74)	0.77	2.7 (1.9–3.5)	0.21
Elevated cTnT	0.44 (0.27–0.70)		2.9 (2.3–3.9)	
Normal NT-proBNP	0.49 (0.32–0.75)	0.86	2.8 (1.9–3.7)	0.89
Elevated NT-proBNP	0.55 (0.24–0.70)		2.5 (2.0–3.5)	
No rhythm disturbances or ischemia	0.49 (0.32–0.71)	0.56	2.7 (1.9–3.8)	0.98
Rhythm disturbances or ischemia	0.49 (0.24–0.74)		2.7 (1.7–3.7)	
Normal QTc	0.45 (0.28–0.67)	0.49	2.5 (1.9–3.4)	0.11
QTc prolongation	0.53 (0.28–0.80)		2.8 (2.3–3.9)	
LVEF > 50%	0.49 (0.28–0.74)	0.92	2.6 (1.8–3.8)	0.41
LVEF ≤ 50%	0.56 (0.33–0.61)		2.0 (1.3–3.5)	
No regional wall motion disturbance	0.49 (0.28–0.74)	0.52	2.7 (1.9–3.8)	0.14
Regional wall motion disturbance	0.33		1.3	
**Neurological outcome, n = 131**				
Independent	0.48 (0.32–0.71)	0.82	2.6 (1.7–3.6)	0.12
Dependent/dead	0.50 (0.24–0.70)		2.8 (2.3–4.1)	

Patients with norepinephrine infusion (n = 65) were excluded from the analyses.

*cTnT* cardiac troponin T, *GCS* Glasgow coma scale, *LVEF* left ventricular ejection fraction, *NT-proBNP* N‐terminal pro‐B‐type natriuretic peptide, *QTc* corrected QT interval.

Concentrations of NT-proBNP were associated with the Glasgow Coma Scale, QTc prolongation and regional wall disturbances on the echocardiography. Concentrations of endogenous catecholamines were similar across all analyzed subgroups, apart from different concentrations of endogenous epinephrine according to the Fisher grading score (lower in more severe grades) and different concentrations of endogenous norepinephrine according to Hunt and Hess and the World Federation of Neurological Surgeons grading score (higher in more severe grades).

### Predictors of neurological outcomes

Table 8 outlines variables indicated by the univariate analysis to be associated with poor neurological outcomes, i.e., dependence (mRS > 2 or GOSE < 6) after SAH.

**Table 8:** Predictors of neurological outcome.

	Independent (n = 102)	Dependent/dead (n = 82)	p
Male sex, n (%)	43 (42.2%)	39 (47.6%)	0.46
Age, years	51.0 (45.0–60.0)	59.0 (49.0–67.0)	< 0.001
Current smoking, n (%)	40 (39.2%)	35 (42.7%)	0.54
Coronary artery disease, n (%)	1 (1.0%)	9 (11.0%)	0.006
Diabetes mellitus, n (%)	5 (4.9%)	7 (8.5%)	0.38
Chronic renal disease,	1 (1.0%)	0 (0.0%)	1.00
Aneurysmal bleed, n (%)	82 (80.4%)	80 (97.6%)	< 0.001
WFNS 1, n (%)	41 (40.2%)	13 (15.9%)	< 0.001
WFNS 2	31 (30.4%)	18 (22.0%)	
WFNS 3	15 (14.7%)	11 (13.4%)	
WFNS 4	9 (8.8%)	32 (39.0%)	
WFNS 5	6 (5.9%)	8 (9.8%)	
SDH	3 (2.9%)	5 (6.1%)	0.47
ICH	11 (10.8%)	32 (39.0%)	< 0.001
Hydrocephalus	43 (42.2%)	50 (60.1%)	0.012
IVH	22 (21.6%)	39 (46.3%)	< 0.001
Any rhythm disturbance, n (%)	13 (12.7%)	14 (17.1%)	0.53
Signs of cardiac ischemia, n (%)	5 (6.1%)	11 (16.4%)	0.06
Elevated cTnT, n (%)	18 (17.6%)	29 (35.4%)	0.005
Elevated NT-proBNP, n (%)	18 (17.6%)	23 (22.5%)	0.07
Norepinephrine, nmol/L	3.1 (2.0–6.0)	7.8 (2.8–28.4)	< 0.001
GCS at arrival	15.0 (14.0–15.0)	12.0 (7.0–15.0)	< 0.001
Worst GCS during the first 24 h	14.0 (11.0–15.0)	10.0 (5.0–14.0)	< 0.001

*cTnT* cardiac troponin T, *GCS* Glasgow coma scale, *ICH* intracerebral hemorrhage, *IVH* intraventricular hemorrhage, *NT-proBNP* N‐terminal pro‐B‐type natriuretic peptide, *QTc* corrected QT interval, *SDH* subdural hemorrhage, *WFNS* World Federation of Neurological Surgeons.

Variables significantly associated with the poor outcome were analyzed further in a multivariable logistic regression model. In this analysis, age (OR 1.04 [95% CI 1.01–1.08]) and the presence of an intracerebral hemorrhage (OR 4.96 [95% CI 1.96–12.60]) and an intraventricular hemorrhage (OR 3.14 [95% CI 1.39–7.11]) were independently associated with poor neurological outcomes. Our model showed an explanatory rate (Nagelkerke) of R^2^ 0.30.

**Figure 1:**
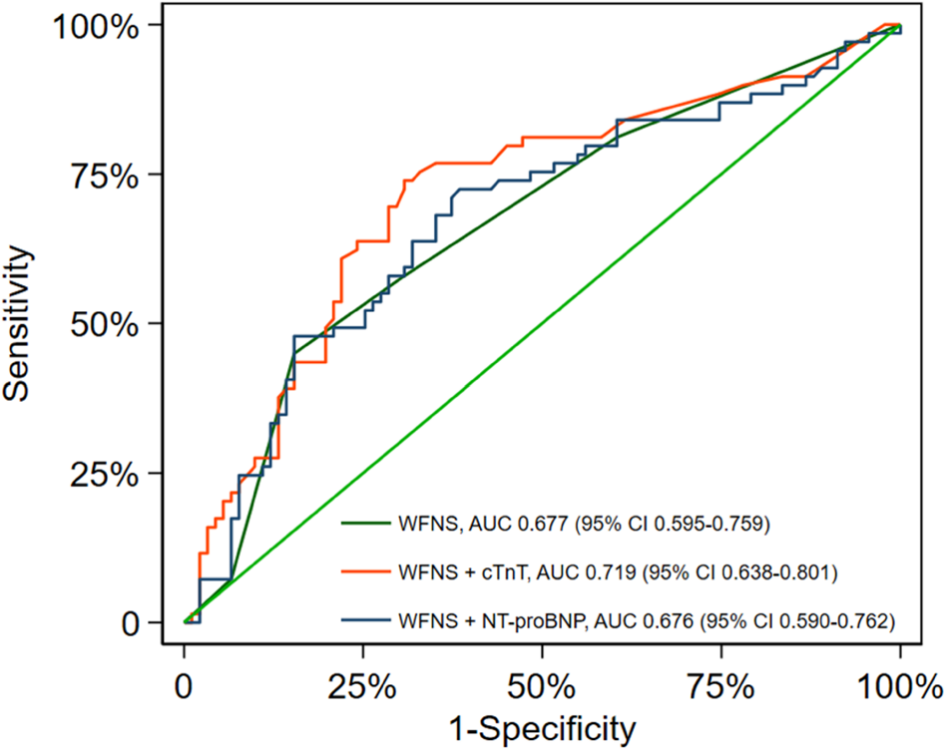
ROC curves for the WFNS grading score, cTnT and NT-proBNP in predicting poor neurological outcomes.

There was a significant association in the logistic regression model between the WFNS grading score, cTnT and poor neurological outcomes at the three-month follow-up (Supplement Table S3). Patients with high WFNS grading scores and cTnT above the median had more than a fourfold increased risk of poor neurological outcomes (adjusted odds ratio 4.45 [95% CI 1.5–13.4]). The area under the receiver operating characteristic curve (ROC-AUC) of the WFNS grading score in predicting poor neurological outcomes was 0.677 (95% CI 0.595–0.759). The addition of cTnT improved the prognostic model of ROC-AUC to 0.719 (95% CI 0.638–0.801), p for comparison = 0.05, Fig. 1). We observed no improvement in cNRI (0.113 [95% CI − 0.188 to 0.473]) or IDI (0.034 [95% CI − 0.005 to 0.107]) when adding cTnT to the WFNS grading score.

Supplement Table S4 shows the associations between the WFNS grading score, NT-proBNP and poor neurological outcomes at the three-month follow-up. Compared to cTnT, the results for NT-proBNP were less consistent, and an association of a high WFNS grading score and a NT-proBNP above the median with poor neurological outcomes was attenuated in the adjusted models. The addition of NT-proBNP to the WFNS grading score did not improve the ROC-AUC for the prognostic model (ROC-AUC 0.68 [95% CI 0.59–0.76], p for comparison = 0.53, Fig. 1). We observed no improvement in cNRI (0.05 [95% CI − 0.40 to 0.40]) or IDI (− 0.004 [95% CI − 0.01 to 0.05]) when adding NT-proBNP to the WFNS grading score.

## Discussion

In a large cohort of patients with non-traumatic SAH, we found no demographic or SAH-related factors associated with cardiac dysfunction at 90 days. The most frequent cardiac findings were increased concentrations of cTnT and NT-proBNP as well as QTc prolongation; however, the overall incidence of cardiac dysfunction was modest. SAH patients with the most severe disease, as quantified by the WFNS grading score and elevated concentrations of cTnT, had an especially poor prognosis at follow-up 90 days after hospital admission.

Cardiac dysfunction appears early and is most often reversible. The left ventricular systolic dysfunction was modest in our study cohort; this finding is in concordance with the findings of the M Tanabe group^12,13^. Diastolic dysfunction is associated with increased troponin^12^, but in our population, diastolic dysfunction was extremely rare. Elevated concentrations of cardiac troponin I are associated with regional wall motion abnormalities in patients with SAH^14^, which is in line with the results from the current investigation, where patients with regional wall motion disturbances exhibited highly elevated concentrations of cTnT. This was also the case for patients with decreased LVEF and QTc prolongation. Furthermore, concentrations of cTnT increased with increasing disease severity quantified by the WFNS grading score. These findings highlight that there is a subpopulation of patients with potential for an early detrimental cardiac impact of SAH, resulting in both overt and subclinical myocardial injury as well as arrhythmia.

Cardiac troponin concentrations are elevated in patients with SAH^14^ and correlate positively with the severity of bleeding^4^, delayed cerebral ischemia, poor outcomes, and mortality^15^. Concentrations of cTnT in our study were uniformly increased according to disease severity in the SAH patients with poor neurological outcomes after 90 days as well. Our results are in concordance with prior studies^14,16^, although elevated cardiac troponin concentrations were less common than in the study by Nastasovic et al.^17^; however, in previous investigations, patients with a known history of cardiac and neurologic diseases have been excluded^18^, making the results less comparable.

Cardiac troponins and natriuretic peptides are the established biomarkers of contemporary cardiology, reflecting myocardial injury and stress. Of the two, cTnT is more strongly associated with disease severity and neurological outcomes. In the absence of an overt myocardial infarction, cardiac troponins are hypothesized to reflect subclinical myocardial injury. The causes of a cardiac troponin increase are multifactorial, possibly including both myocardial ischemia and strain. In our patients with acute non-traumatic SAH, the activation of the renin–angiotensin, sympathetic and inflammatory systems may have mediated the cardiac troponin release. In comparison, NT-proBNP and catecholamines are less frequently associated with cardiac complications, disease severity and poor neurological outcomes.

Elevated catecholamine concentrations are used as surrogate markers for increased sympathetic activity^19^. Our study results are in concordance with results from the Moussoutas group^20^, where norepinephrine levels but not epinephrine levels were associated with clinical status. Salem et al.^21^ showed that myocardial alterations and catecholamine concentrations are regressive during the first week, which is comparable to the findings of our study. We measured catecholamine concentrations at the same time points as the cardiac echocardiography and ECGs were performed but found no association of cardiac function and arrhythmia with endogenous catecholamine concentrations.

Our study has its strengths and limitations. We included study patients from three different hospitals in two European countries with a high quality of neuro-intensive care. A major strength is the repeated multimodal cardiac assessment with a long-term follow-up. The incidence of morbidity and cardiac complications was modest compared to previous studies. One explanation is that our population reflects a whole spectrum of non-traumatic SAH, not only selected poor-grade patients.

## Conclusion

Patients with non-traumatic SAH are at risk for cardiac complications, especially with regard to a subclinical myocardial injury and arrhythmia. Along with clinical risk scoring systems, measurements of cardiac troponin may improve risk assessments for long-term prognosis. There could be a subgroup of patients, who should be multidisciplinary evaluated at day 90, to identify those in need of cardiac care.

## Supplementary Information

Supplementary Information.

## Data Availability

The datasets generated and/or analyzed during the current study are not publicly available due the Finnish legislation. The applicable Finnish legislations does not allow sharing and/or submitting datasets from the study. Applicable legislation states that 1) any health data should only be processed if there is a valid legal basis. Even then, any transfer to a country outside EU/EEA requires specific basis and protective measures. (General Data Protection Regulation, GDPR) 2) patient data is strictly confidential and cannot be revealed to third parties (Act on the Status and Rights of Patients) 3) secondary use of health data (e.g., for scientific research) must comply with the Act on Secondary use of Data. This legislation prohibits us from allowing data transfers/ access to datasets that would include patient/health data. Therefore, raw data or even de-identified (pseudonymized) data cannot be shared publicly. The datasets used and/or analyzed during the current study are available from the corresponding author on reasonable request.

## Abbreviations

Ao: Aortic root diameter
AV: Atrioventricular
BMI: Body mass index
BNP: B-type natriuretic peptide
CI: Confidence Interval
CK: Creatinine kinase
CK-MB: Creatinine kinase myocardial band
cNRI: Continuous net reclassification index
CRP: C-reactive protein
DT: Deceleration time
cTnT: Cardiac troponine T
E/Eʹ: By mitral peak E-wave velocity by peak Eʹ velocity
ECG: Electrocardiogram
eGFR: Estimated glomerular filtration
EGTA: Ethylene glycolbis (2-aminoethylether)-N, N, Nʹ, Nʹ-tetra acetic acid
GCS: Glasgow coma scale
GOSE: Glasgow outcome scale extended
E/A: E-wave and A-wave velocities
ECLIA: Electrochemiluminescence immunoassay
IDI: Integrated discrimination improvement
INR: International normalized ratio
ICH: Intracerebral hemorrhage
IVH: Intraventricular hemorrhage
ICU: Intensive care unit
IVS: Interventricular wall thickness
IQR: Interquartile range
Lat Eʹ: Lateral mitral annulus
LA: Left atrium diameter
LV: Left ventricle
LVEDD: LV end-diastolic diameter
LVESD: LV end-systolic diameter
LVEF: Left ventricular ejection fraction
MRs: Modified Rankin scale
NT-ProBNP: N‐terminal Pro‐B‐type natriuretic peptide
PW: Posterior wall thickness
QTc: Corrected QT interval
SAH: Subarachnoid hemorrhage
SDH: Subdural hemorrhage
Sept Eʹ: Septal annulus
SDNN: Standard deviation of RR-interval
TAPSE: Tricuspid annulus plane systolic excursion
TTE: Transthoracic echocardiography
WFNS: World federation of neurological surgeons
