# Supplementary material for: The prevalence of cardiac complications and their impact on outcomes in patients with non-traumatic subarachnoid hemorrhage
Source: Sci Rep. 2022 Nov 22;12:20109. doi: 10.1038/s41598-022-24675-8 (PMC9684538; doi:10.1038/s41598-022-24675-8)
Supplement: Supplementary file 1 — Supplementary Information. [file 41598_2022_24675_MOESM1_ESM.pdf]

The prevalence of cardiac complications and their impact on outcomes in patients with non-traumatic subarachnoid hemorrhage.

Maarit Lång, MD, Kuopio University Hospital, Department of Intensive Care Medicine, Finland

Stephan M Jakob, MD, PhD, Professor, Department of Intensive Care Medicine, Bern University Hospital, University of Bern, Bern, Switzerland

Riikka Takala MD, PhD, Associate Professor, Perioperative Services, Intensive Care Medicine and Pain Management, Turku University Hospital, Anaesthesiology, Intensive Care, Emergency Care and Pain Medicine, University of Turku, Finland

Magnus N Lyngbakken, MD, PhD, Associate Professor, Department of Cardiology, Division of Medicine, Akershus University Hospital, Lørenskog, Institute of Clinical Medicine, University of Oslo, Oslo, Norway

Anu Turpeinen, MD, PhD, Associate Professor, Kuopio University Hospital, Department of Cardiology, Finland

Torbjørn Omland, MD, PhD, Professor, Department of Cardiology, Division of Medicine, Akershus University Hospital, Lørenskog, Institute of Clinical Medicine, University of Oslo, Oslo, Norway

Tobias M Merz, MD *, Department of Intensive Care Medicine, Bern University Hospital, University of Bern, Bern, Switzerland, *Current affiliation: Cardiothoracic and Vascular Intensive Care Unit, Auckland City Hospital, Auckland, New Zealand

Jan Wiegand, MD [+], Department of Intensive Care Medicine, Bern University Hospital, University of Bern, Bern, Switzerland, [+]Current affiliation: Intensive Care Unit, Lindenhofspital, Bern, Switzerland.

Juha Grönlund, MD PhD, Perioperative Services, Intensive Care Medicine and Pain Management, Turku University Hospital, Anaesthesiology, Intensive Care, Emergency Care and Pain Medicine, University of Turku, Finland

Melissa Rahi, MD, PhD, Neurocenter, Department of Neurosurgery, Turku University Hospital, Finland

Mika Valtonen, MD PhD, Associate Professor, Perioperative Services, Intensive Care Medicine and Pain Management, Turku University Hospital, Anaesthesiology, Intensive Care, Emergency Care and Pain Medicine, University of Turku, Finland

Timo Koivisto, MD, PhD, Associate Professor, Kuopio University Hospital, Department of Neurosurgery, Finland

Helge Røsjø, MD, PhD, Professor, Division for Research and Innovation, Akershus University Hospital, Lørenskog, Institute of Clinical Medicine, University of Oslo, Oslo, Norway

Stepani Bendel, MD, PhD, Associate Professor 1. Kuopio University Hospital, Department of Intensive

Correspondence:

Maarit Lång, MD

Kuopio University Hospital

PO BOX 100, 70029 KYS, Finland

E-mail: maarit.lang@kuh.fi

Key Words: subarachnoid haemorrhage, cardiac failure, cardiac dysfunction, Tnt, myocardial injury, outcome

SUPPLEMENT TABLE 1. Baseline characteristics according to WFNS grading score for SAH						
	WFNS 1	WFNS 2	WFNS 3	WFNS 4	WFNS 5	
**N**	55	53	26	42	16	p for trend
Clinical characteristics						
Male sex, n (%)	29 (52.7%)	20 (37.7%)	10 (38.5%)	19 (45.2%)	7 (43.8%)	0.58
Age, years	54.0 (42.0-63.0)	52.0 (47.0-59.0)	59.0 (48.0-65.0)	57.5 (50.0-65.0)	55.0 (45.5-63.0)	0.24
Current smoking, n (%)	20 (38.5%)	24 (47.1%)	12 (52.2%)	18 (47.4%)	4 (26.7%)	0.49
Body mass index, kg/m^2	26.3 (23.8-28.7)	25.7 (23.1-28.7)	26.4 (22.9-29.9)	27.7 (24.5-29.4)	24.7 (23.7-27.7)	0.38
Systolic blood pressure, mmHg	138.5 (123.0-149.0)	136.5 (128.0-150.0)	138.0 (122.0-151.0)	128.5 (118.0-146.0)	134.0 (123.0-145.0)	0.31
Diastolic blood pressure, mmHg	62.5 (54.0-71.0)	59.0 (52.0-66.0)	57.0 (48.0-65.0)	60.0 (51.0-65.0)	58.0 (54.5-70.5)	0.41
Medical history						
Hypertension, n (%)	15 (27.3%)	12 (22.6%)	9 (34.6%)	18 (42.9%)	2 (12.5%)	0.12
Coronary artery disease, n (%)	0 (0.0%)	2 (3.8%)	3 (11.5%)	4 (9.5%)	1 (6.3%)	0.06
Hypercholesterolemia, n (%)	4 (7.3%)	2 (3.8%)	1 (3.8%)	3 (7.1%)	1 (6.3%)	0.93
Atrial fibrillation, n (%)	0 (0.0%)	0 (0.0%)	1 (3.8%)	1 (2.4%)	0 (0.0%)	0.28
Ischemic stroke, n (%)	3 (5.5%)	2 (3.8%)	0 (0.0%)	1 (2.4%)	0 (0.0%)	0.88
Diabetes mellitus, n (%)	3 (5.5%)	4 (7.5%)	2 (7.7%)	3 (7.1%)	0 (0.0%)	0.92
Chronic renal disease, n (%)	1 (1.8%)	0 (0.0%)	0 (0.0%)	0 (0.0%)	1 (6.3%)	0.22
Electrocardiography						
Corrected QT time, ms	428.0 (419.5-457.5)	445.0 (416.0-475.0)	447.0 (432.0-464.0)	446.0 (428.0-495.0)	467.5 (445.0-534.5)	0.014

Any rhythm disturbance, n (%)	11 (20.0%)	7 (13.2%)	3 (11.5%)	6 (14.3%)	0 (0.0%)	0.38
Signs of ischemia, n (%)	2 (4.5%)	3 (6.8%)	4 (17.4%)	5 (14.7%)	2 (16.7%)	0.24
Any rhythm disturbance or ischemia, n (%)	12 (21.8%)	9 (17.0%)	7 (26.9%)	10 (23.8%)	2 (12.5%)	0.75
First degree AV block, n (%)	8 (15.7%)	8 (15.4%)	3 (11.5%)	13 (31.7%)	0 (0.0%)	0.05
Echocardiography						
LV ejection fraction, %	64.0 (58.0-70.0)	61.5 (55.0-70.0)	60.0 (57.0-67.0)	68.0 (60.0-72.0)	60.5 (54.0-73.0)	0.42
E/A ratio	1.3 (1.0-1.7)	1.3 (1.1-1.7)	1.2 (1.0-1.6)	1.1 (0.9-1.5)	1.2 (1.0-1.5)	0.29
e'	10.9 (9.0-13.6)	11.0 (9.0-12.0)	11.0 (9.0-13.0)	10.0 (7.0-12.0)	9.0 (7.9-10.3)	0.21
E/e’	7.6 (6.0-8.8)	7.8 (6.6-9.0)	7.9 (7.0-8.2)	7.6 (6.3-9.1)	8.2 (6.2-8.5)	0.87
TAPSE, mm	27.0 (25.0-30.0)	26.0 (23.0-29.0)	24.0 (21.0-27.0)	26.0 (20.1-29.5)	24.0 (21.0-26.0)	0.11
Regional wall motion disturbance, n (%)	0 (0.0%)	2 (3.8%)	1 (3.8%)	3 (7.1%)	2 (12.5%)	0.10
Radiography						
Pneumonia, n (%)	1 (7.7%)	2 (8.3%)	1 (8.3%)	2 (8.7%)	2 (12,5%)	0.44
Congestion, n (%)	0 (0.0%)	0 (0.0%)	1 (8.3%)	0 (0.0%)	0 (0.0%)	
Clinical chemistry						
eGFR, ml/min/1.73m^2	108.9 (102.7-116.3)	109.3 (98.3-117.6)	104.9 (95.5-110.6)	99.3 (92.4-112.6)	103.2 (86.1-111.0)	0.13
Epinephrine, nmol/L	0.5 (0.3-0.7)	0.5 (0.3-0.8)	0.3 (0.1-0.6)	0.2 (0.1-0.4)	0.5 (0.3-0.7)	0.003
Norepinephrine, nmol/L	2.6 (1.7-3.8)	3.5 (2.3-7.4)	5.9 (3.2-9.2)	17.7 (3.8-45.3)	10.2 (2.6-33.8)	<0.001
Lactate, mmol/L	1.2 (0.9-1.4)	1.2 (0.9-1.6)	1.4 (1.1-2.4)	1.8 (1.3-2.2)	1.8 (1.3-3.1)	<0.001
CRP, mg/L	4.0 (1.5-11.0)	7.0 (3.0-13.0)	20.0 (7.5-32.0)	15.0 (7.0-37.0)	28.0 (9.0-69.0)	<0.001

cTnT, ng/L	5.5 (2.5-10.0)	6.0 (2.5-13.0)	9.0 (2.5-13.0)	12.5 (7.0-26.5)	151.5 (22.0-375.0)	<0.001
NT-proBNP, ng/L	311.5 (196.0-565.0)	456.0 (215.0-680.0)	300.0 (146.0-910.0)	410.0 (236.0-1058.0)	1183.0 (340.0-2310.0)	0.06
Clinical outcomes						
ICU stay, days	2.0 (1.0-5.0)	4.0 (2.0-9.0)	10.0 (5.0-13.0)	9.0 (6.0-13.0)	10.5 (5.5-15.0)	<0.001
Hospital stay, days	11.0 (9.0-14.0)	13.0 (10.0-18.0)	14.5 (11.0-22.0)	14.0 (9.0-20.0)	14.0 (9.0-20.5)	0.05
ICU mortality, n (%)	1 (1.8%)	1 (1.9%)	0 (0.0%)	4 (9.5%)	2 (12.5%)	0.07
3 month mortality, n (%)	1 (1.9%)	1 (2.0%)	0 (0.0%)	12 (29.3%)	4 (28.6%)	<0.001
Poor neurological outcome, n (%)	13 (24.1%)	18 (36.7%)	11 (42.3%)	32 (78.0%)	8 (57.1%)	<0.001

Continuous data are presented as median (IQR) and categorical data as number (proportion). AV, atrioventricular. CRP, C-reactive protein. cTnT, cardiac troponin T. eGFR, estimated glomerular filtration rate. GCS, Glasgow coma scale. ICU, intensive care unit. LV, left ventricular. NT-proBNP, N-terminal pro-B-type natriuretic peptide. QTc, corrected QT interval. TAPSE, tricuspid annulus plane systolic excursion.

SUPPLEMENT TABLE 2. **Predictive factors of decreased LVEF at 90 days**

**N=126**	EF≤50%<br>n=22 (17.5%)	D90 EF>50%<br>n=104 (82.5%)	p
Age, years	53.1±10.0	54.4±13.2	0.64
Gender, n (%)			
Male	9 (40.9%)	44 (42.3%)	0.90
Female	13 (59.1%)	60 (57.7%)	
BMI	26.3±4.7	26.7±4.6	0.72
Aneurysm location, n (%)	n=19	n=103	
ICA	4 (21.1%)	21 (20.4%)	0.82
MCA	5 (26.3%)	25 (24.3%)	
ACoA	6 (31.5%)	34 (33.0%)	
ACA	0	7 (6.8%)	
VBA	4 (21.1%)	16 (15.5%)	
No aneurysm, n (%)	3 (13.6%)	0	<0.05
Hunt Hess, n (%)	n=20	n=92	
1-2 (headache)	9 (45.0%)	54 (58.7%)	0.37
3 (drowsy)	4 (20.0%)	19 (20.7%)	

4-5 (stupor/coma)	7 (35.0%)	19 (20.7%)	
Fisher scale, n (%)<br>1 (No blood, thin)<br>2 (Clots)<br>3 (Diffuse ICH, IVH)	n=21<br>5 (23.8%)<br>8 (38.1%)<br>8 (38.1%)	n=102<br>18 (17.6%)<br>41 (40.2%)<br>43 (42.2%)	 0.80
SDH, n (%)	1 (4.5%)	4 (3.8%)	0.88
ICH, n (%)	3 (13.6%)	21 (20.2%)	0.56
Hydrocephalus, n (%)	9 (40.9%)	50 (48.1%)	0.54
IVH, n (%)	5 (22.7%)	28 (26.9%)	0.68
Cardiovascular disease, n (%)	6 (27.3%)	28 (26.9%)	0.97
Neurologic disease, n (%)	4 (18.2%)	10 (9.8%)	0.26
Pulmonary disease, n (%)	2 (9.1%)	11 (10.6%)	0.84
Diabetes, n (%)	2 (9.1%)	3 (2.9%)	0.18
Chronic renal disease, n (%)	1 (4.5%)	1 (1.0%)	0.22
Smoker, n(%)<br>Ex-smoker	9 (40.9%)<br>2 (9.1%)	49 (47.1%)<br>10 (9.6%)	0.92

No smoking	10 (45.5%)	39 (37.5%)	
Drug abuse (alcohol, drugs), n (%)	3 (13.6%)	7 (6.7%)	0.53
ECG rhythm disturbances<br><br>at day 1, n (%)	5 (22.7%)	16 (15.4%)	0.40
ECG ischemia at day 1, n (%)	4 (18.2%)	7 (6.7%)	0..08
Tnt ng/L at day 1	9 [5-17]	7 [5-14]	0.60
ProBNB ng/L at day 1	375.0 [156.5-985.0]	406.0 [241.0-727.0]	0.75
Endogenous P-norepinephrine consentration nmol/L at day1	1.44 [1.1-3.39]	2.66 [1.88-3.56]	0.23
GCS at arrival, n (%)<br><br>Good (9-15)<br><br>Bad (3-8)	n=22<br><br>18 (81.8%)<br><br>4 (18.2%)	n=101<br><br>86 (85.1%)<br><br>15 (14.9%)	 <br>0.70
Worst GCS during the first 24 hours, n (%)<br><br>Good (9-15)<br><br>Bad (3-8)	n=22<br><br>12 (57.1%)<br><br>10 (42.9%)	n=102<br><br>83 (81.4%)<br><br>19 (18.6%)	 <br>0.02
WFNS, n (%)<br><br>1	 <br>4 (18.2%)	 <br>31 (30.1%)	 <br>0.50

2	8 (36.4%)	35 (33.7%)	
3	2 (9.1%)	16 (15.4%)	
4	6 (27.3%)	15 (14.4%)	
5	2 (9.1%)	7 (6.7%)	
GOSE dependent, n (%)	4 (19.0%)	24 (25.3%)	0.55
mRS dependent, n (%)	3 (15.0%)	15 (16.9%)	0.84

SD=standard deviation and IQR= interquartile range. ICA: internal cerotic artery, MCA: middle cerebral artery, ACoA: anterior communicating artery, ACA: anterior cerebral artery, VBA: vertebrobasilar artery. SDH: subdural hematoma, ICH: intracerebral hematoma, IVH: intraventricular hematoma; ECG: electrocardiogram, Tnt; troponin_t, ProBNB: B-type N-terminal peptide, ECHO: cardiac ultrasound, GCS: Glascow coma scale; WFNS; World Federation of Neurosurgical Societies

SUPPLEMENT TABLE 3. Association of WFNS score and cTnT with poor neurological outcome

	Odds ratio (95% CI)				
	WFNS - cTnT -	WFNS - cTnT +	WFNS + cTnT -	WFNS + cTnT +	P for trend
Model 1	1.00	1.75 (0.71 to 4.31)	1.73 (0.63 to 4.77)	6.33 (2.71 to 14.80)	<0.001
Model 2	1.00	1.16 (0.44 to 3.06)	1.66 (0.59 to 4.70)	5.08 (2.10 to 12.29)	<0.001
Model 3	1.00	0.86 (0.25 to 2.94)	2.00 (0.58 to 6.95)	4.45 (1.45 to 13.36)	0.006

WFNS -, < 3. WFNS +, ≥ 3. cTnT -, < 8 ng/L. cTnT +, ≥ 8 ng/L. Model 1, unadjusted. Model 2, adjusted for age and sex. Model 3, adjusted for model 2, systolic blood pressure, BMI, coronary artery disease, diabetes mellitus, current smoking, eGFR, and concentrations of norepinephrine. BMI, body mass index. eGFR, estimated glomerular filtration rate.

SUPPLEMENT TABLE 4. Association of WFNS score and NT-proBNP with poor neurological outcome

	Odds ratio (95% CI)				
	WFNS - NT-proBNP -	WFNS - NT-proBNP +	WFNS + NT-proBNP -	WFNS + NT-proBNP +	P for trend
Model 1	1.00	1.26 (0.52 to 3.03)	3.85 (1.48 to 9.98)	3.19 (1.30 to 7.84)	0.002
Model 2	1.00	0.86 (0.33 to 2.22)	3.06 (1.13 to 8.27)	2.66 (1.02 to 6.92)	0.007
Model 3	1.00	0.59 (0.18 to 1.87)	3.97 (1.20 to 13.2)	1.49 (0.43 to 5.17)	0.10

WFNS -, < 3. WFNS +, ≥ 3. NT-proBNP -, NT-proBNP < 380 ng/L. NT-proBNP +, NT-proBNP ≥ 380 ng/L. Model 1, unadjusted. Model 2, adjusted for age and sex. Model 3, adjusted for model 2, systolic blood pressure, BMI, coronary artery disease, diabetes mellitus, current smoking, eGFR, and concentrations of norepinephrine.
